# Contribution of Mouse Embryonic Stem Cells and Induced Pluripotent Stem Cells to Chimeras through Injection and Coculture of Embryos

**DOI:** 10.1155/2014/409021

**Published:** 2014-12-28

**Authors:** Jitong Guo, Baojiang Wu, Shuyu Li, Siqin Bao, Lixia Zhao, Shuxiang Hu, Wei Sun, Jie Su, Yanfeng Dai, Xihe Li

**Affiliations:** ^1^Research Center for Animal Genetic Resources of Mongolia Plateau, Inner Mongolia University, Hohhot 010021, China; ^2^Inner Mongolia Saikexing Reproductive Biotechnology Co., Ltd., Helingeer 011517, China

## Abstract

Blastocyst injection and morula aggregation are commonly used to evaluate stem cell pluripotency based on chimeric contribution of the stem cells. To assess the protocols for generating chimeras from stem cells, 8-cell mouse embryos were either injected or cocultured with mouse embryonic stem cells and induced pluripotent stem cells, respectively. Although a significantly higher chimera rate resulted from blastocyst injection, the highest germline contribution resulted from injection of 8-cell embryos with embryonic stem cells. The fully agouti colored chimeras were generated from both injection and coculture of 8-cell embryos with embryonic stem cells. Additionally, microsatellite DNA screening showed that the fully agouti colored chimeras were fully embryonic stem cell derived mice. Unlike embryonic stem cells, the mouse chimeras were only generated from injection of 8-cell embryos with induced pluripotent stem cells and none of these showed germline transmission. The results indicated that injection of 8-cell embryos is the most efficient method for assessing stem cell pluripotency and generating induced pluripotent stem cell chimeras, embryonic stem cell chimeras with germline transmission, and fully mouse embryonic stem cell derived mice.

## 1. Introduction

Chimeric mice are important tools for investigating embryonic development as they can provide insights into the function of a specific gene, they can trace the origin of the cell lineage, and they can assess the potential of cells. The first chimeric mouse was produced in 1961 [1] through the aggregation of two 8-cell embryos. In later years, chimeric mice were created by microinjections of dissociated inner-cell-mass cells (ICMs) into the cavity of a blastocyst [2]. Since then, the techniques and protocols have been modified and improved [3–6] such that chimeric mice have been produced successfully using embryos aggregated or injected with ICM cells [2], embryonal carcinoma cells (ECCs) [7], embryonic stem cells (ESCs) [8], embryonic germ cells (EGCs) [9], somatic cell nuclear transfer-derived embryonic stem cells (ntESCs) [10], induced pluripotent stem cells (iPSCs) [11, 12], spermatogonial stem cells (SSCs) [13, 14], extraembryonic endoderm (XEN) cells [15], and epiblast stem cells (EpiSCs) [16, 17]. Chimeras are a mixture of cells derived from both the donor cells and those of the recipient embryo. Since it is extremely difficult to determine the chimeric contribution of donor cells in particular tissues, chimeras are usually identified by means of coat coloration.

Conventional injection of ESCs into a blastocyst is the most popular method of producing chimeras and those generated are partially derived from the ESCs. Well sandwich aggregation is also a highly stable and reproducible method for generating germline transmitted chimeras, but two embryos are required to effect the procedure [6, 18]. Although microinjection will also produce good germline transmitted chimeras, specialized equipment is needed. For these reasons, the coculture method was developed to produce chimeric mice [3, 4, 19] even though it is far less efficient than the microinjection and well sandwich aggregation techniques. However, an improved method for producing chimeras with a high degree of chimerism and germline transmission which utilized coculture of denuded mouse embryos and ES cells in Eppendorf tubes was reported [20]. Such aggregation in Eppendorf tubes can cause embryo adhesion and form 2- or 3-embryo clusters mixed with ESCs.

Recently, 8-cell stage embryos were used to generate fully ESC-derived mice in a laser- [21] and piezo-assisted micromanipulation system [22]. The authors determined that this 8-cell method is effective for both inbred ES cells, such as C57BL/6 and 129, and hybrid ESCs to generate fully ESC-derived mice; thus, F0-generation mice enable immediate phenotyping [22]. ESCs can adhere to blastomeres and migrate into the ICM after microinjection or aggregation. Nevertheless, the mechanism of ESC migration in chimeric embryos remains unclear [23–25] and how they can completely replace the ICM of an embryo and develop into an ESC-derived mouse demands investigation. Hence, in the present study we evaluated ESC development potential and protocols for chimera generation in the production of fully ESC-derived mice. While ESCs are recognized as pluripotent stem cells, we also tested the chimeric contributions of iPSCs on chimera generation by microinjection and coculture in the well-of-the-well (WOW) system.

## 2. Materials and Methods

### 2.1. Materials

#### 2.1.1. Animals

KM albino mice purchased from Vital River (Beijing, China) were housed at 20–24°C with 12 h of light and 12 h of darkness. All the experiments were carried out according to the regulatory guidelines for experimental animals approved by the State Council of China.

#### 2.1.2. Reagents

All the chemicals used were purchased from Sigma-Aldrich (USA) unless otherwise stated. Equine chorionic gonadotropin (eCG) and human chorionic gonadotropin (hCG) were purchased from the Ningbo Sansheng Pharmaceutical Co., Ltd., China.

### 2.2. Methods

#### 2.2.1. Culture of ESCs and iPSCs

Mouse ESC lines were isolated from the outgrowths of day 3.5 blastocysts derived from 129/Sv females mated to Oct4-ΔPE-GFP transgenic males as described [6, 26]. Transgenic green fluorescent protein (GFP) expression by the reporter gene was under the control of the Oct4 promoter and distal enhancer, while the proximal enhancer region was deleted. This GFP transgene showed expression in the ICM of blastocysts, PGCs* in vivo,* and ESCs [27]. ESCs were maintained in knockout Dulbecco's modified Eagle's medium (DMEM, Gibco) containing 15% fetal bovine serum (FBS, Gibco), 2 mM GlutaMAX, 0.1 mM 2-mercaptoethanol (Gibco), 1% penicillin-streptomycin (Gibco), 1% MEM nonessential amino acids (Gibco), and 1000 U/mL leukaemia inhibitory factor (LIF, ProSpec) on mitomycin C-treated mouse embryonic fibroblast (MEF) feeder cells.

Mouse iPSCs were derived from Rex1-GFP mouse MEFs (129xMF1) [28] reprogrammed by the PiggyBac transposon carrying* Oct4*,* Sox2*,* Klf4*, and* cMyc* reprogramming vectors [29]. The iPSCs were maintained in knockout DMEM (Gibco) containing 15% FBS (Gibco), 2 mM GlutaMAX, 0.1 mM 2-mercaptoethanol (Gibco), 1% penicillin-streptomycin (Gibco), 1% MEM nonessential amino acids (Gibco), and 1000 U/mL LIF (ProSpec) on mitomycin C-treated STO feeder cells.

Mouse ESCs and iPSCs were then frozen in FBS plus 10% dimethyl sulfoxide (DMSO). For cell injection and aggregation, thawed cells were cultured without feeder cells on a 0.1% gelatin-treated 12-well cell culture plate (Corning) and used within 4 days. Before experiments, cells were trypsinized with 0.025% trypsin-EDTA (Gibco) and resuspended in ESC-maintaining medium.

#### 2.2.2. Collection of Mouse Embryos

KM albino female mice 6–8 weeks old were superovulated by intraperitoneal injection of 5 IU eCG and 5 IU hCG at 17:00, 48 h apart. At the time of the hCG injection, the females were mated by KM males. Vaginal plugs were checked the following morning when the time was defined as day 0.5 post coitum (dpc). Females with vaginal plugs were killed by cervical dislocation at 2.5 and 3.5 dpc for collection of, respectively, 8-cell embryos and blastocysts. The oviducts or uteri were removed and transferred into 20 mM HEPES-buffered KSOM (95 mM NaCl, 2.5 mM KCl, 0.35 mM KH_2_PO_4_, 0.2 mM MgSO_4_ *·*7H_2_O, 0.2 mM D-glucose, 10 mM Na-lactate, 4 mM NaHCO_3_, 0.2 mM Na pyruvate, 1.71 mM CaCl*·*2H_2_O, 1.0 mM glutamine, 0.01 mM Na_2_EDTA*·*2H_2_O, and 1% penicillin and streptomycin) [30] supplemented with 3.0 mg/mL bovine serum albumin (BSA) in 35 mm Petri dishes (Corning). They were then flushed with a fine bore needle attached to a 1 mL syringe [6]. The 8-cell embryos were retrieved and cultured in 4-well dishes (Nunc) in KSOM modified with 25 mM NaHCO_3_ and supplemented with 1.0 mg/mL BSA, 0.5% MEM nonessential amino acids (Gibco), and 0.5% essential amino acids (Gibco) at 37°C in a humidified atmosphere containing 5% CO_2_ before being used subsequently in experiments. Blastocysts were used immediately for ESC injection.

#### 2.2.3. Generation of Mouse Chimeras by Injection of Embryos with ESCs and iPSCs

Approximately 10–15 cells were aspirated into the injection pipette and injected gently into the blastocoel cavity using a piezo-assisted micromanipulator attached to an inverted microscope [22, 31]. The injected embryos were cultured to enable reexpansion of the blastocoel cavity and then transferred to the uteri of pseudopregnant KM mice at 2.5 dpc [6].

In a similar manner to the injection of blastocysts, 8-cell embryos were injected with cells placed carefully into the perivitelline space under the zona pellucida. The injected embryos were cultured overnight; blastocysts that developed were transferred to the uteri of pseudopregnant KM mice at 2.5 dpc [6]. Chimeras were confirmed by the coat color pattern of the pups at birth.

#### 2.2.4. Generation of Mouse Chimeras by Coculture of 8-Cell Embryos with ESCs and iPSCs

The zona pellucida was removed from 8-cell embryos by brief exposure to Tyrode's solution. The denuded embryos were then washed with HEPES-buffered KSOM and transferred into 4-well dishes in KSOM in one of the WOW systems [32]. Approximately 100 cells were selected and transferred into each well of the system to coculture them with the embryo overnight. The resulting blastocysts were transferred to the uteri of pseudopregnant KM mice at 2.5 dpc [6]. Again, chimeras were identified by coat color of the pups at birth.

#### 2.2.5. Embryo Transfer

Embryo transfer recipients were prepared by pairing mature KM female mice with vasectomized KM males overnight. Vaginal plugs were examined the following morning and plugged mice were used as pseudopregnant recipients for embryo transfer. They were anesthetized by intraperitoneal injection of Avertin (0.3 mg/g body weight) and 10–15 blastocysts were transferred into the tip of each uterine horn.

#### 2.2.6. Microsatellite DNA Analysis of Fully ESC-Derived Mice

Chimeric pups were initially identified by coat color, and the full agouti-coated mice were selected for microsatellite DNA analysis [33]. Sequences of microsatellite marker primers were obtained from the Mouse Genome Informatics website (The Jackson Laboratory, http://www.informatics.jax.org) [34, 35] (Table 1). Genomic DNA was extracted from tail biopsies recovered from the chimeric mice and from recipient and donor ESCs using a DNeasy Blood & Tissue Kit (Qiagen). Microsatellite DNAs were amplified by polymerase chain reaction (PCR) using a Type-it Microsatellite PCR Kit (Qiagen). The PCR reaction was performed in a 25 *μ*L volume containing 1 *μ*g DNA, 10 *μ*mol/L primers, and 12.5 *μ*L double strength master mix. Amplification was performed in 96-well microtiter plates and the PCR conditions were initialized at 94°C for 30–60 sec: 25–40 cycles of denaturation at 94°C for 30–60 sec, annealing at 55°C for 30–60 sec, and extension at 72°C for 1 min, followed by a final extension step at 72°C for 5–10 min (Table 1). PCR products were diluted (1 : 1) in loading buffer and electrophoresed in 12% polyacrylamide gels at 150 V for 4 h [36]. The resulting gels were then silver stained, scanned, and photographed [37]. Briefly, the gel was fixed and stained for 5 min in a solution containing 5% ethanol, 1% HNO_3_, and 0.1% AgNO_3_ and then washed in water. DNA bonds were developed for 8 min in developer containing 1.3% NaOH, 0.65% NaCO_3_, and 0.4% formaldehyde. Development was then stopped by addition for 1 min of 5% ethanol with 1% HNO_3_. The gels were stored in water for photography.

#### 2.2.7. Transmission Screening of Mouse Chimeras

Some chimeras and fully ESC-derived mice were selected and mated with KM mice. The germline transmission competence of ESCs and iPSCs was determined by the coat colors of the resulting F1 pups.

### 2.3. Statistical Analysis

Data expressed as percentages were analyzed using the chi-squared test (http://statpages.org/ctab2x2.html). A *P* value less than 0.05 between two groups within the same column was considered to indicate significance.

## 3. Results

### 3.1. Generation of Mouse Chimeras Using ESCs

**Figure 1:**
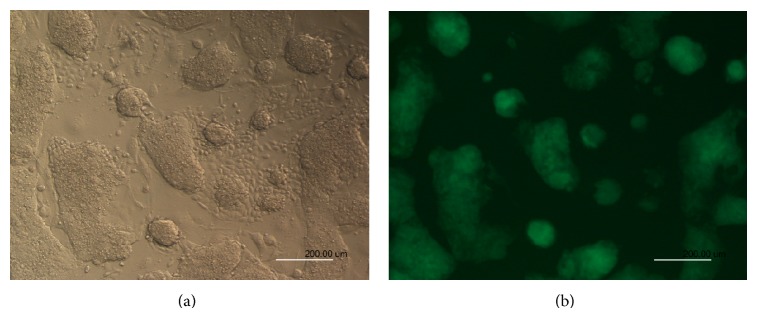
ESC and iPSC cultures without feeder cells. ES cells were cultured on day 3 after thawing: bright field (a) and GFP fluorescence (b).

**Figure 2:**
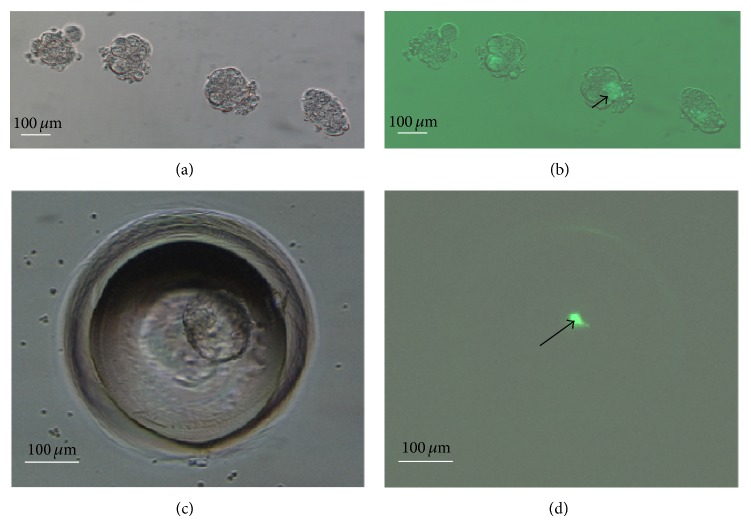
Generation of mouse chimeras by 8-cell embryo coculture with ESCs. Morulae developed from ESC coculture ((a) bright field; (b) GFP fluorescence); the arrow shows that ESCs were aggregated into the recipient embryos. Blastocysts developed from ESC coculture in the WOW system ((c) bright field; (d) GFP fluorescence). The arrow shows how ESCs were aggregated into the ICM of recipient embryos.

**Figure 3:**
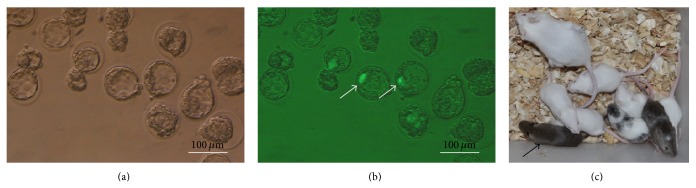
Generation of mouse chimeras by injection of ESCs into 8-cell embryos. Blastocysts developed from ES cell injection ((a) bright field; (b) GFP fluorescence). The white arrow shows that ESCs were aggregated into the ICM of blastocysts. Chimeric mice generated by injecting ESCs into 8-cell embryos (c). The black arrow shows the full agouti-colored chimeric mouse.

When ESCs at passage 20–22 were used for chimera production (Figure 1), 29.1% (25/86), 18.1% (23/127), and 38.4% (28/73) of the pups derived from, respectively, 8-cell embryo coculture (Figure 2), 8-cell embryo injection (Figures 3(a) and 3(b)), and blastocyst injection showed coat color chimerism (Table 2). The chimeric rate from blastocyst injection was significantly higher than that from 8-cell embryo injection, but there was no significant difference between the 8-cell embryo injection and 8-cell embryo coculture groups. Three of 25 (12.0%) and one of 23 (4.3%) full-colored chimeras were produced by 8-cell embryo injection and 8-cell coculture, respectively (Figure 3(c)). No full-colored chimeras were produced by blastocyst injection.

### 3.2. Generation of Mouse Chimeras Using iPSCs

**Figure 4:**
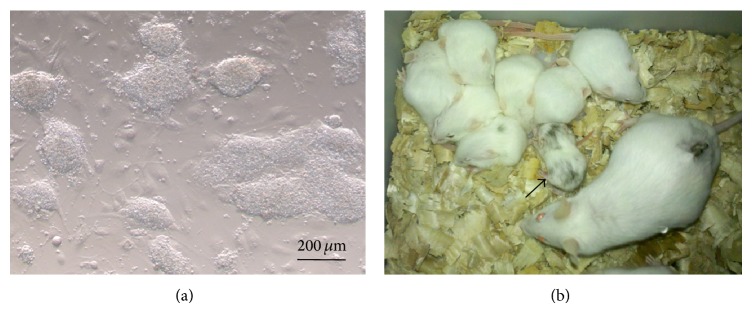
Generation of mouse chimeras with iPSCs iPS. Cells were cultured on day 3 after thawing (a). Chimeric mice generated by injection of 8-cell embryos with iPSCs (b). The black arrow shows the chimeric mouse with the colored coat.

Chimeric pups (13.8%, 8/58) were produced only by injection of iPSCs at passage 10–12 in 8-cell embryos (Figure 4), although nonchimeric pups were produced in the 8-cell coculture and blastocyst injection groups (Table 3). Most iPSC chimeras were fertile when mated with female KM mice, although no germline transmitting chimeras were produced.

### 3.3. Microsatellite DNA Analysis of Fully ESC-Derived Mice

**Figure 5:**
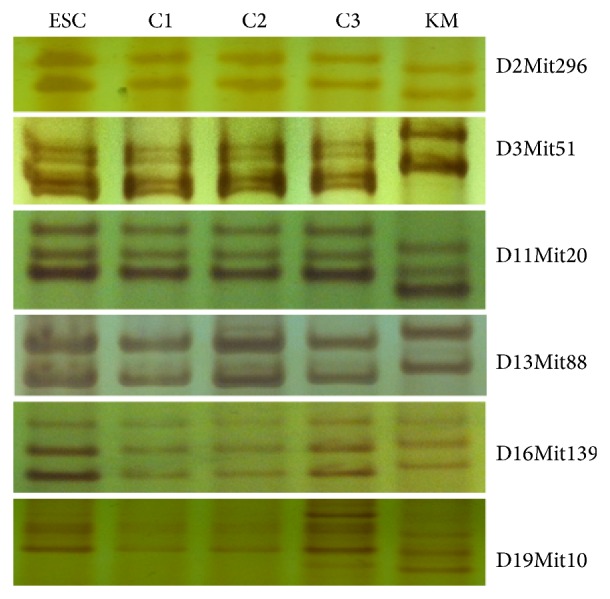
Microsatellite DNA analysis of fully ESC-derived mice. Microsatellite DNAs from cultured ESCs, three chimeric mice (C1, C2, and C3), and KM mouse recipient were amplified. Microsatellite loci D2Mit296, D3Mit51, D11Mit20, D13Mit88, D16Mit139, and D19Mit10 are shown.

Three full agouti-colored chimeras were scanned for microsatellite DNAs to determine whether they were fully ESC-derived mice. This showed that all 3 mice were fully derived from ESCs, but not from KM mouse embryos (Figure 5).

### 3.4. Germline Transmission of Mouse Chimeras Derived from ESCs

**Figure 6:**
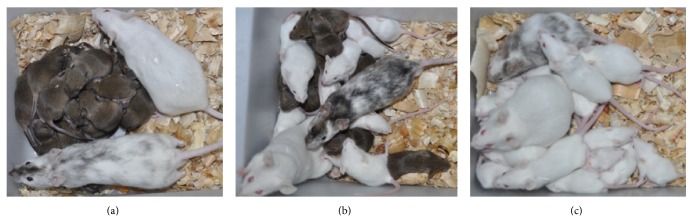
Germline transmission of mouse chimeras derived from ESCs. Fully (a), partially (b), and no (c) germline transmitted chimeric mice are shown by the colors of their pups.

The highest germline contribution from ESCs was observed in the 8-cell embryo injection group (69.2%, 9/13). This compared to 45.5% (5/11) for the 8-cell coculture and 8.3% (1/12) for the blastocyst injection groups, respectively (Table 4). No significant differences were observed in the percentages of fertile chimeras among the 8-cell embryo coculture (78.6%, 11/14), 8-cell embryo injection (86.7%, 13/15), and blastocyst injection (92.3%, 12/13) groups (Table 3). However, no germline chimeras were produced from iPSCs. Germline transmission of the chimeras was assessed by the colors of the pups. The results showed that the percentage of coat colors derived from ESCs was not predictive of germline transmission (Figure 6).

## 4. Discussion

Expression of pluripotency genes, immunocytochemistry of pluripotency markers, embryoid body formation* in vitro,* and teratoma formation* in vivo* are generally used to test the pluripotency of stem cells [38, 39]. More importantly, however, their pluripotency is best evaluated based according to their contribution of cells in chimeras. Blastocyst injection and morula aggregation are the methods of choice to generate stem cell chimeras [3, 6, 40]. Our results are similar to other published reports; more germline transmitted chimeras were produced from the same batch of ESCs by 8-cell embryo injection than by blastocyst injection in our experiments, despite more pups being produced by the latter [41, 42]. Eight-cell embryo injection is physically more demanding than blastocyst injection due to the small size of the perivitelline space increasing the chances of damage to the blastomeres of the embryo during the injection procedure. In addition, the beginning of processes involved in compaction of mouse 8-cell embryos might be disturbed by injection of ESC. Consequently, both the ICM and the trophectoderm are unable to differentiate into normal cell types at the correct time, thereby resulting in loss of pups. This indicates that compaction of the mouse embryo is important for its further development [43] and that coculture of 8-cell embryos and ESCs is more desirable to generate transmitting chimeras from ESCs. We conclude that the aggregation modified by our method of coculture in WOW is more convenient and efficient for generating ESC chimeras and even fully ESC-derived mice compared with that of blastocyst and 8-cell embryo injection [22, 44].

ESC chimeras were generated by 8-cell embryo injection, blastocyst injection, and 8-cell embryo coculture in this study. However, iPSC chimeras could be generated only by 8-cell embryo injection, although none of the resulting iPSC chimeras were germline transmitted. Interestingly, implantation nodules were observed when the embryos derived from 8-cell embryos cocultured with iPSCs were transferred to recipient mice but sections of these implantations showed that the fetuses had been replaced by tumorous cells (data not shown). Thus, we presume that the iPSCs, due to their oncogenicity, drove the ICM cells into tumor rather than embryo formation. iPSC chimeras can be generated by injection of 8-cell embryos because fewer iPSCs were injected into the perivitelline space, unlike the situation in 8-cell embryo coculture. Here, a few iPSCs were induced by ICM cells which then developed into normal embryos. These results suggest that the contribution of iPSCs in chimeras does not correspond to their full pluripotency potential [45].

Our study complements the previous report that formation of the cell niche in chimeric embryos is closely associated with the dominant cells and the cell niche determines the fates of stem cells [46]. The ICM cell niche dominates the ESC's niche, but the iPSC niche is dominant to the ICM. Thus, fewer iPSCs similar to ESCs can be induced to form ICM cell-like cells and contribute to chimeras, but large numbers of iPSCs can induce ICM cells to transform into iPSC-like cells, which ultimately results in tumor formation.

We found that fully ESC-derived mice could be produced by coculturing and injecting 8-cell embryos but not blastocysts and that coculture is a simple and effective method for producing chimeras and fully ESC-derived mice [21, 22, 47]. This improved coculture technique is more convenient than vial coculture [20]. The finding also indicates that the protocol used has little effect on the generation of fully ESC-derived mice, but the embryonic stage has a marked effect. Consistent with other reports, ESCs can adhere to the surfaces of 8-cell embryos, but not to 2- and 4-cell embryos [25]. Although the developmental mechanism of fully ESC-derived mice remains unclear, we presume that ESC clusters tend to form ICM cells due to their tight junctions [48–52] and asymmetry formation derived from variation in cell sizes and shapes [53] between ESCs and blastomeres [51, 54] because more fully ESC-derived mice were generated by coculture of 8-cell embryos and ESCs in which ESC clusters aggregated with embryos were formed. Further investigation is required to determine how ES cells completely replace ICMs and develop thereafter into an ESC-derived mouse.

In conclusion, our noninvasive 8-cell embryo coculture is a simple and suitable protocol for generating ESC chimeras and fully ECS-derived mice that can be employed to characterize the totipotency of ESC lines. However, 8-cell embryo injection to generate iPSC chimeras is the only suitable method for characterizing chimeric developmental potential of iPSCs. The ability of stem cells to contribute to chimeric animals may not represent the totipotency of stem cells. Transmission of stem cells and fully stem-cell-derived animals should also be considered.

## Supplementary Material

The methods and results of characterization of ESCs and iPSCs were summarized in the Supplementary Material. Briefly, mouse ESCs and iPSCs were characterized by immunocytochemical staining for Nanog, Sox2 and Oct4, RT-PCR for Sox2, Oct4, Nanog and Klf4, and teratoma formation. The methods of immunocytochemical staining, RT-PCR and teratoma formation were described in the Supplementary Material. The results showed that the ESCs and iPSCs isolated in our laboratory were typical stem cells.

## Figures and Tables

**Table 1:** Sequences of primers specific for microsatellite markers.

Locus	Primer sequence (5′→3′)	Conditions			
		Denaturation	Annealing	Number of cycles	Extension
D2Mit296	CAACTGTAAATCCAGTCGTAGGG CTCTGCTGAGGTTACTGTGGG	94°C, 30 sec	55°C, 30 sec	40	5 min

D3Mit51	GGCACTGATAGCAGGCCTAG TCTCTTCTGGTATTTCCTTCCG	94°C, 60 sec	55°C, 60 sec	25	10 min

D11Mit20	CCTGTCCAGGTTTGAGAGGA CTTGGGAGCCTCTTCGGT	94°C, 60 sec	55°C, 60 sec	35	10 min

D13Mit88	ACTGATGGCTCATGAGACCC AAAATTAATAGGAACTGCAAGGG	94°C, 30 sec	55°C, 30 sec	40	5 min

D16Mit139	GTATGTAAGGAATGGTCAAATTCTTG TCATTGTGATTGTGAAAGAATGC	94°C, 30 sec	55°C, 30 sec	40	5 min

D19Mit10	GCCTTTAAGCCAGTCAAGACA CCAGTCTGGACTTGTGAATGA	94°C, 3 sec	55°C, 30 sec	40	5 min

**Table 2:** Generation of chimeric mice with ESCs.

Method	Number of embryos transferred	Number of recipients	Number of pregnant recipients (%)	Number of pups	Number of chimeras (%)	
					Total	Full colored
8-cell coculture	312	14	11 (78.6)^a^	86	25 (29.1)^ab^	3 (12.0)^a^
8-cell injection	365	17	14 (82.4)^a^	127	23 (18.1)^a^	1 (4.3)^a^
Blastocyst injection	129	6	6 (100)^a^	73	28 (38.4)^b^	0 (0.0)^a^

Total	806	37	31 (83.8)	286	76 (26.6)	4 (1.4)

^a,b^Values with different superscripts within the same column are significantly different (*P* < 0.05).

**Table 3:** Generation of chimeric mice using iPSCs.

Method	Number of embryos transferred	Number of recipients	Number of pregnant recipients (%)	Number of pups	Number of chimeras (%)
8-cell coculture	341	16	1 (6.3)^a^	1	0 (0)
8-cell injection	192	11	10 (90.9)^b^	58	8 (13.8)^a^
Blastocyst injection	287	14	9 (64.3)^b^	83	0 (0)^b^

Total	820	41	20 (48.8)	142	8 (5.6)

^a,b^Values with different superscripts within the same column are significantly different (*P* < 0.05).

**Table 4:** Germline-transmitting chimeras produced from ESCs.

Method	Germline transmission		
	Tested	Fertile (%)	Germline (%)
8-cell coculture	14	11 (78.6)^a^	5 (45.5)^ab^
8-cell injection	15	13 (86.7)^a^	9 (69.2)^a^
Blastocyst injection	13	12 (92.3)^a^	1 (8.3)^b^

Total	42	36 (85.7)^a^	15 (75.0)

^a,b^Values with different superscripts within the same column are significantly different (*P* < 0.05).

## References

[1] Tarkowski A. K. (1961). Mouse chimæras developed from fused eggs. *Nature*.

[2] Gardner R. L. (1968). Mouse chimaeras obtained by the injection of cells into the blastocyst. *Nature*.

[3] Wood S. A., Allen N. D., Rossant J., Auerbach A., Nagy A. (1993). Non-injection methods for the production of embryonic stem cell-embryo chimaeras.. *Nature*.

[4] Wood S. A., Pascoe W. S., Schmidt C., Kemler R., Evans M. J., Allen N. D. (1993). Simple and efficient production of embryonic stem cell-embryo chimeras by coculture. *Proceedings of the National Academy of Sciences of the United States of America*.

[5] Moustafa L. A., Brinster R. L. (1972). Induced chimaerism by transplanting embryonic cells into mouse blastocysts. *Journal of Experimental Zoology*.

[6] Nagy A., Gertsenstein M., Vintersten K., Behringer R. (2003). *Manipulating the Mouse Embryo: A Laboratory Manual*.

[7] Mintz B., Illmensee K. (1975). Normal genetically mosaic mice produced from malignant teratocarcinoma cells. *Proceedings of the National Academy of Sciences of the United States of America*.

[8] Bradley A., Evans M., Kaufman M. H., Robertson E. (1984). Formation of germ-line chimaeras from embryo-derived teratocarcinoma cell lines. *Nature*.

[9] Matsui Y., Zsebo K., Hogan B. L. M. (1992). Derivation of pluripotential embryonic stem cells from murine primordial germ cells in culture. *Cell*.

[10] Wakayama T., Tabar V., Rodriguez I., Perry A. C. F., Studer L., Mombaerts P. (2001). Differentiation of embryonic stem cell lines generated from adult somatic cells by nuclear transfer. *Science*.

[11] Okita K., Ichisaka T., Yamanaka S. (2007). Generation of germline-competent induced pluripotent stem cells. *Nature*.

[12] Takahashi K., Yamanaka S. (2006). Induction of pluripotent stem cells from mouse embryonic and adult fibroblast cultures by defined factors. *Cell*.

[13] Kanatsu-Shinohara M., Inoue K., Lee J., Yoshimoto M., Ogonuki N., Miki H., Baba S., Kato T., Kazuki Y., Toyokuni S., Toyoshima M., Niwa O., Oshimura M., Heike T., Nakahata T., Ishino F., Ogura A., Shinohara T. (2004). Generation of pluripotent stem cells from neonatal mouse testis. *Cell*.

[14] Kanatsu-Shinohara M., Lee J., Inoue K. (2008). Pluripotency of a single spermatogonial stem cell in mice. *Biology of Reproduction*.

[15] Kunath T., Arnaud D., Uy G. D., Okamoto I., Chureau C., Yamanaka Y., Heard E., Gardner R. L., Avner P., Rossant J. (2005). Imprinted X-inactivation in extra-embryonic endoderm cell lines from mouse blastocysts. *Development*.

[16] Brons I. G. M., Smithers L. E., Trotter M. W. B., Rugg-Gunn P., Sun B., Chuva De Sousa Lopes S. M., Howlett S. K., Clarkson A., Ahrlund-Richter L., Pedersen R. A., Vallier L. (2007). Derivation of pluripotent epiblast stem cells from mammalian embryos. *Nature*.

[17] Tesar P. J., Chenoweth J. G., Brook F. A., Davies T. J., Evans E. P., Mack D. L., Gardner R. L., McKay R. D. G. (2007). New cell lines from mouse epiblast share defining features with human embryonic stem cells. *Nature*.

[18] Hogan B. (1994). *Manipulating the Mouse Embryo: A Laboratory Manual*.

[19] Shimada H., Kaname T., Suzuki M. (1999). Comparison of ES cell fate in sandwiched aggregates and co-cultured aggregates during blastocyst formation by monitored GFP expression. *Molecular Reproduction and Development*.

[20] Lee K.-H., Chuang C.-K., Wang H. W., Stone L., Chen C.-H., Tu C.-F. (2007). An alternative simple method for mass production of chimeric embryos by coculturing denuded embryos and embryonic stem cells in Eppendorf vials. *Theriogenology*.

[21] Poueymirou W. T., Auerbach W., Frendewey D. (2007). F0 generation mice fully derived from gene-targeted embryonic stem cells allowing immediate phenotypic analyses. *Nature Biotechnology*.

[22] Huang J., Deng K., Wu H., Liu Z., Chen Z., Cao S., Zhou L., Ye X., Keefe D. L., Liu L. (2008). Efficient production of mice from embryonic stem cells injected into four-or eight-cell embryos by piezo micromanipulation. *Stem Cells*.

[23] Johnson M. H., Ziomek C. A. (1981). Induction of polarity in mouse 8-cell blastomeres: specificity, geometry, and stability. *The Journal of Cell Biology*.

[24] Saburi S., Azuma S., Sato E., Toyoda Y., Tachi C. (1997). Developmental fate of single embryonic stem cells microinjected into 8-cell-stage mouse embryos. *Differentiation*.

[25] de Repentigny Y., Kothary R. (2010). Production of mouse chimeras by injection of embryonic stem cells into the perivitelline space of one-cell stage embryos. *Transgenic Research*.

[26] Tang F., Barbacioru C., Bao S. (2010). Tracing the derivation of embryonic stem cells from the inner cell mass by single-cell RNA-seq analysis. *Cell Stem Cell*.

[27] Yeom Y. I. I., Fuhrmann G., Ovitt C. E., Brehm A., Ohbo K., Gross M., Hübner K., Schöler H. R. (1996). Germline regulatory element of Oct-4 specific for the totipotent cycle of embryonal cells. *Development*.

[28] Wang W., Yang J., Liu H., Lu D., Chen X., Zenonos Z., Campos L. S., Rad R., Guo G., Zhang S., Bradley A., Liu P. (2011). Rapid and efficient reprogramming of somatic cells to induced pluripotent stem cells by retinoic acid receptor gamma and liver receptor homolog 1. *Proceedings of the National Academy of Sciences of the United States of America*.

[29] Yusa K., Rad R., Takeda J., Bradley A. (2009). Generation of transgene-free induced pluripotent mouse stem cells by the piggyBac transposon. *Nature Methods*.

[30] Erbach G. T., Lawitts J. A., Papaioannou V. E., Biggers J. D. (1994). Differential growth of the mouse preimplantation embryo in chemically defined media. *Biology of Reproduction*.

[31] Kawase Y., Iwata T., Watanabe M., Kamada N., Ueda O., Suzuki H. (2001). Application of the piezo-micromanipulator for injection of embryonic stem cells into mouse blastocysts. *Contemporary Topics in Laboratory Animal Science*.

[32] Vajta G., Peura T. T., Holm P. (2000). New method for culture of zona-included or zona-free embryos: the Well of the Well (WOW) system. *Molecular Reproduction and Development*.

[33] Zhang X., Zhu Z., Huang Z., Tan P., Ma R. Z. (2007). Microsatellite genotyping for four expected inbred mouse strains from KM mice. *Journal of Genetics and Genomics*.

[34] Matouk C., Gosselin D., Malo D., Skamene E., Radzioch D. (1996). PCR-analyzed microsatellites for the inbred mouse strain 129/Sv, the strain most commonly used in gene knockout technology. *Mammalian Genome*.

[35] Simpson E. M., Linder C. C., Sargent E. E., Davisson M. T., Mobraaten L. E., Sharp J. J. (1997). Genetic variation among 129 substrains and its importance for targeted mutagenesis in mice. *Nature Genetics*.

[36] Guilliatt A. M. (2002). Agarose and polyacrylamide gel electrophoresis. *Methods in Molecular Biology*.

[37] An Z. W., Xie L. L., Cheng H., Zhou Y., Zhang Q., He X. G., Huang H. S. (2009). A silver staining procedure for nucleic acids in polyacrylamide gels without fixation and pretreatment. *Analytical Biochemistry*.

[38] Adewumi O., Aflatoonian B., Ahrlund-Richter L. (2007). Characterization of human embryonic stem cell lines by the international stem cell initiative. *Nature Biotechnology*.

[39] Martí M., Mulero L., Pardo C., Morera C., Carrió M., Laricchia-Robbio L., Esteban C. R., Belmonte J. C. I. (2013). Characterization of pluripotent stem cells. *Nature Protocols*.

[40] Carstea A. C., Pirity M. K., Dinnyes A. (2009). Germline competence of mouse ES and iPS cell lines: chimera technologies and genetic background. *World Journal of Stem Cells*.

[41] Tokunaga T., Tsunoda Y. (1992). Efficacious production of viable germ-line chimeras between embryonic stem (ES) cells and 8-cell stage embryos. *Development Growth and Differentiation*.

[42] Yagi T., Tokunaga T., Furuta Y. (1993). A novel ES cell line, TT2, with high germline-differentiating potency. *Analytical Biochemistry*.

[43] Saiz N., Plusa B. (2013). Early cell fate decisions in the mouse embryo. *Reproduction*.

[44] Ramírez M. A., Fernández-González R., Pérez-Crespo M., Pericuesta E., Gutiérrez-Adán A. (2009). Effect of stem cell activation, culture media of manipulated embryos, and site of embryo transfer in the production of F0 embryonic stem cell mice. *Biology of Reproduction*.

[45] Tachibana M., Sparman M., Ramsey C., Ma H., Lee H.-S., Penedo M. C. T., Mitalipov S. (2012). Generation of chimeric rhesus monkeys. *Cell*.

[46] James D., Noggle S. A., Swigut T., Brivanlou A. H. (2006). Contribution of human embryonic stem cells to mouse blastocysts. *Developmental Biology*.

[47] DeChiara T. M., Poueymirou W. T., Auerbach W., Frendewey D., Yancopoulos G. D., Valenzuela D. M. (2010). Producing fully ES cell-derived mice from eight-cell stage embryo injections. *Methods in Enzymology*.

[48] Plusa B., Frankenberg S., Chalmers A., Hadjantonakis A.-K., Moore C. A., Papalopulu N., Papaioannou V. E., Glover D. M., Zernicka-Goetz M. (2005). Downregulation of Par3 and aPKC function directs cells towards the ICM in the preimplantation mouse embryo. *Journal of Cell Science*.

[49] Fleming T. P., Garrod D. R., Elsmore A. J. (1991). Desmosome biogenesis in the mouse preimplantation embryo. *Development*.

[50] Johnson M. H., Maro B., Takeichi M. (1986). The role of cell adhesion in the synchronization and orientation of polarization in 8-cell mouse blastomeres. *Journal of Embryology and Experimental Morphology*.

[51] Morris S. A., Teo R. T. Y., Li H., Robson P., Glover D. M., Zernicka-Goetz M. (2010). Origin and formation of the first two distinct cell types of the inner cell mass in the mouse embryo. *Proceedings of the National Academy of Sciences of the United States of America*.

[52] Zernicka-Goetz M. (2004). First cell fate decisions and spatial patterning in the early mouse embryo. *Seminars in Cell and Developmental Biology*.

[53] Ziomek C. A., Johnson M. H. (1980). Cell surface interaction induces polarization of mouse 8-cell blastomeres at compaction. *Cell*.

[54] Ducibella T., Anderson E. (1975). Cell shape and membrane changes in the eight cell mouse embryo: prerequisites for morphogenesis of the blastocyst. *Developmental Biology*.

