# Supplementary material for: Contribution of Mouse Embryonic Stem Cells and Induced Pluripotent Stem Cells to Chimeras through Injection and Coculture of Embryos
Source: Stem Cells Int. 2014 Dec 28;2014:409021. doi: 10.1155/2014/409021 (PMC4291195; doi:10.1155/2014/409021)
Supplement: Supplementary file 1 — The methods and results of characterization of ESCs and iPSCs were summarized in the Supplementary Material. Briefly, mouse ESCs and iPSCs were characterized by immunocytochemical staining for Nanog, Sox2 and Oct4, RT-PCR for Sox2, Oct4, Nanog and Klf4, and teratoma formation. The methods of immunocytochemical staining, RT-PCR and teratoma formation were described in the Supplementary Material. The results showed that the ESCs and iPSCs isolated in our laboratory were typical stem cells. [file 409021.f1.pdf]

Supplementary Materials

Supplementary methods

Immunocytochemistry of mouse ESCs and iPSCs

Immunocytochemical staining of mouse ESCs and iPSCs using Nanog, Sox2, and Oct4 antibodies was performed following the staining procedure recommended by Abcam [1]. Briefly, the cells were passaged on MEF feeder cells growing on 12-well glass slides (MP Biomedicals) before being fixed in 4% paraformaldehyde and blocked with 1% BSA. The fixed cells were then incubated with rabbit anti-Nanog (Abcam; 1:100), rabbit anti-Sox2 (Abcam; 1:250) and rabbit anti-Oct4 (Abcam; 1:250) antibodies overnight at 4 °C. After washing, the cells were incubated with goat anti-rabbit IgG conjugated with FITC (Abcam; 1:500) for 1 h at room temperature in the dark. Before mounting under Vectashield medium (Vector Laboratories), the cells were incubated with 1 μg/mL DAPI for 1 min. Finally, the cells were covered by coverslips which were sealed around the perimeter for prolonged storage. The slides were examined and evaluated for fluorescence using an Olympus FluoView FV1000 microscope.

RT-PCR of mouse ESCs and iPSCs

Mouse ESCs and iPSCs were subjected to RT-PCR using the Sox2, Oct4, Nanog, and Klf4 primers. Briefly, total RNA was extracted from the cells using an RNeasy Mini Kit (QIAGEN) and reverse transcription was performed using a Reverse Transcription System Kit (Promega, USA). PCR was performed using a Taq PCR Master Mix Kit (QIAGEN) according to the

manufacturer's protocols. The primer sequences used for PCR are detailed in Supplemented Table 1. The PCR reaction was performed in a volume of 12.5 μL containing 2 μL cDNA and each primer at 10 μM. It ran for 30 cycles at an initial 4 min denaturation, and then 30 sec denaturation at 94 ℃, 30 sec annealing, and 40 sec extension at 72 ℃ for 30 cycles. The reaction was terminated by a final elongation step of 5 min at 72 ℃, and the products were visualized on a 1.5% (w/v) agarose gel stained with ethidium bromide and photographed under GelDoc-It (UVP).

Teratoma formation from mouse ESCs and iPSCs

Mouse ESCs and iPSCs at approximately 70% confluence were harvested and dissociated into single cell suspensions using 0.05% Trypsin/EDTA (Gibco). The cells were washed with PBS (Gibco) and filtered through a 40 μm cell strainer. The filtered cells were then injected into the hind legs of NOD-SCID mice at a dose of 2×10^6 cells/site. An injection of feeder cells was used as the negative control. Mice exhibiting tumors 6–8 weeks after the cell injections were sacrificed and the tumors were collected in 4% paraformaldehyde. They were then sectioned and stained with hematoxylin and eosin to enable microscopic identification of the types of tissues that comprised each tumor.

Supplementary figures

Figure 1. Immunocytochemical staining of mouse ESCs and iPSCs

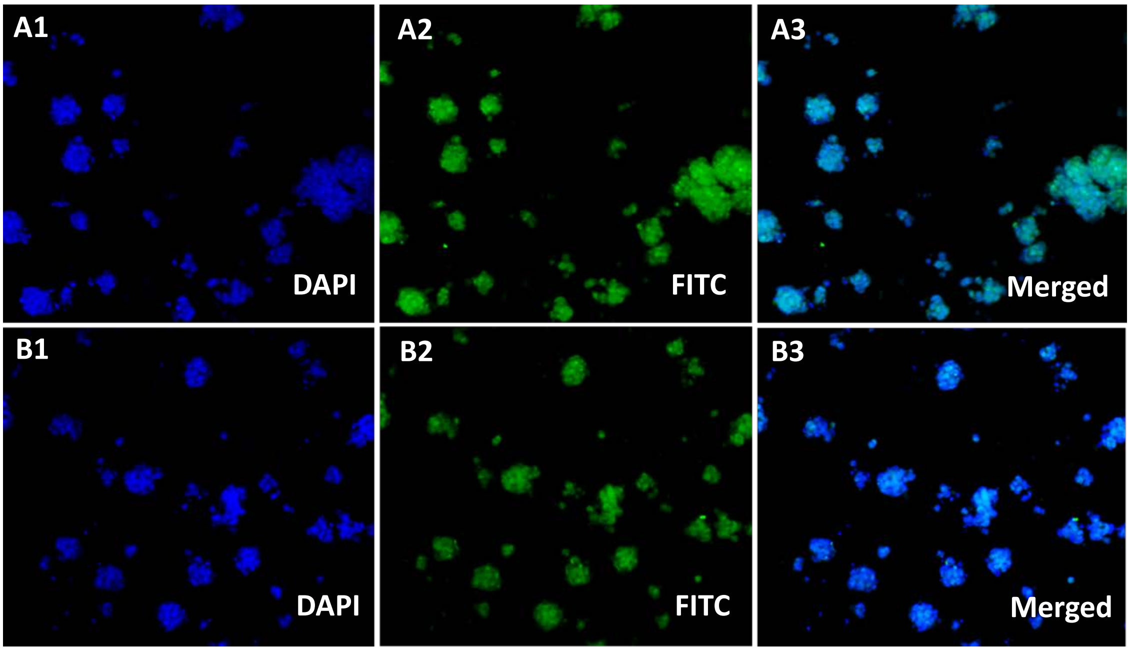

Figure 1A. Immunocytochemical staining of mouse ESCs and iPSCs for Nanog. Mouse ESCs and iPSCs isolated in this study were positive for Nanog. Results for DAPI, FITC, and DAPI merged with FITC are showed in A1 and B1, A2 and B2, and A3 and B3 for ESCs and iPSCs, respectively.

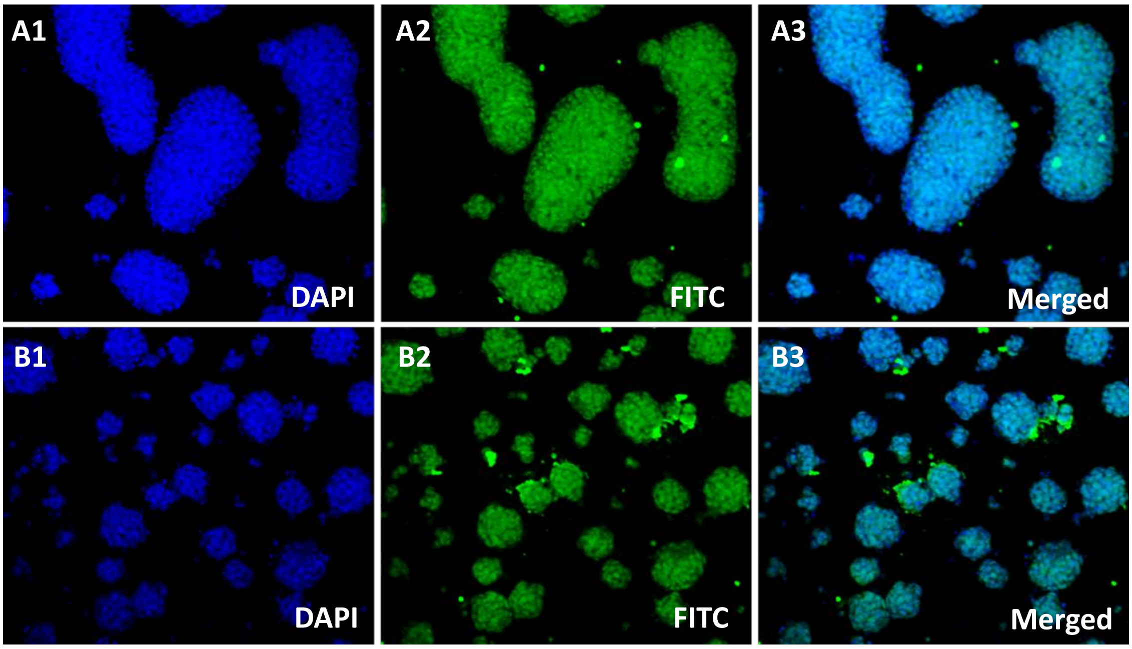

Figure 1B. Immunocytochemical staining of mouse ESCs and iPSCs for Sox2.

Mouse ESCs and iPSCs isolated in the study were positive for Sox2. DAPI, FITC, and DAPI merged with FITC are shown in A1 and B1, A2 and B2, and A3 and B3 for ESCs and iPSCs, respectively.

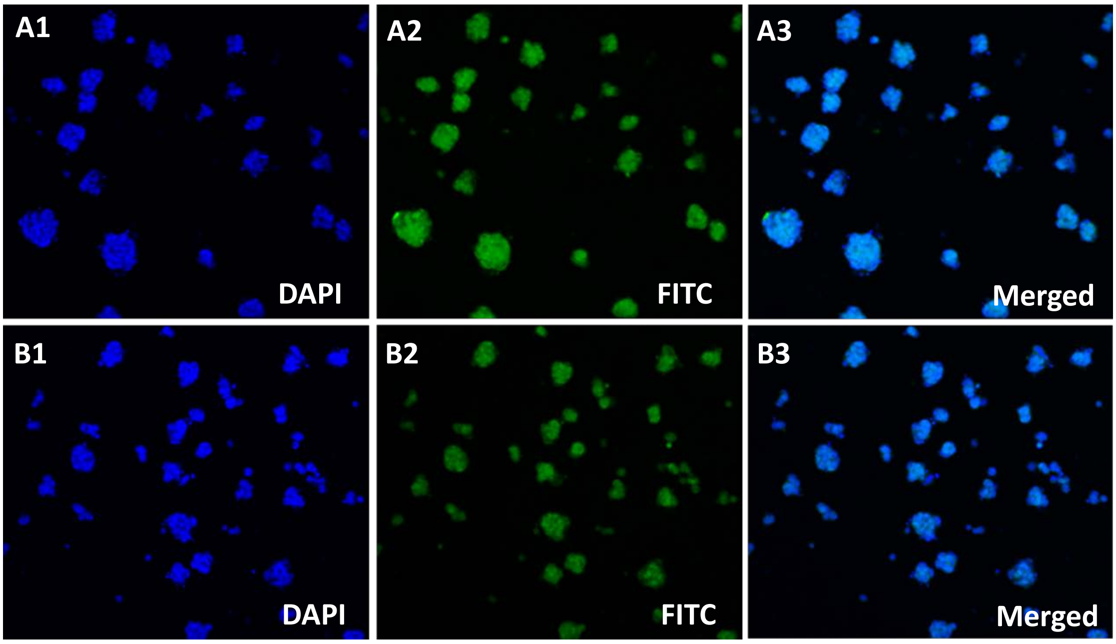

Figure 1C. Immunocytochemical staining of mouse ESCs and iPSCs for Oct4. Mouse ESCs and iPSCs isolated in the study were positive for Oct4. DAPI, FITC, and DAPI merged with FITC are shown in A1 and B1, A2 and B2, and A3 and B3 for ESCs and iPSCs, respectively.

Figure 2. RT-PCR of mouse ESCs and iPSCs

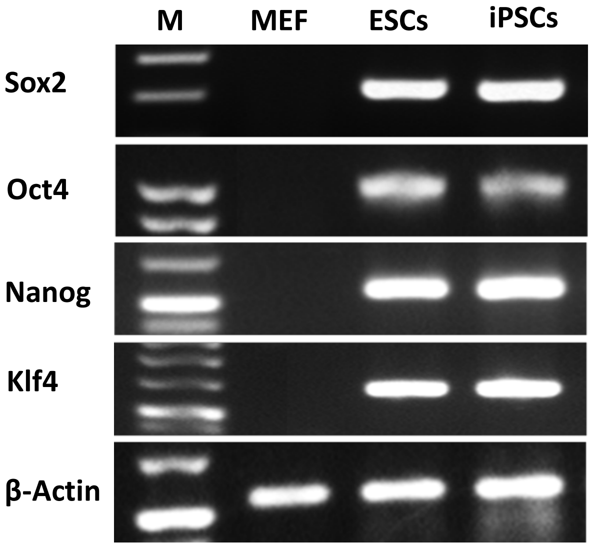

Figure 2. RT-PCR analyses showed that Sox2, Oct4, Nanog, and Klf4 were expressed in mouse ESCs and iPSCs. β-Actin served as a positive control. M = 50bp ladder DNA marker. MEF = mouse embryonic fibroblasts.

Figure 3. Teratoma formation by mouse ESCs and iPSCs

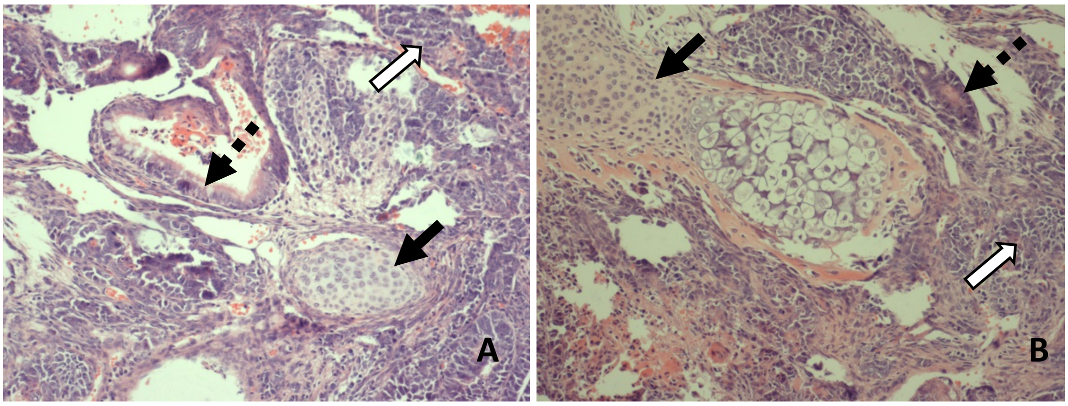

Sections of teratoma derived from mouse ESCs (A) and iPSCs (B). The typical three germ layers are indicated by arrows. White arrows = ectodermal structures, black arrows = mesodermal structures, and dashed arrows = endodermal structures, respectively.

Supplemental table

Table 1. The primer sequences of RT-PCR

Name	Primer	Tm (℃)	Length (bp)
Sox2	GGCAGCTACAGCATGATGCAGGAGC<br>CTGGTCATGGAGTTGTACTGCAGG	64.2	131
Oct4	CTGAGGGCCAGGCAGGAGCACGAG<br>CTGTAGGGAGGGCTTCGGGCACTT	68.9	485
Nanog	CAGGTGTTTGAGGGTAGCTC<br>CGGTTCATCATGGTACAGTC	50.5	223
Klf4	TGCCAGACCAGATGCAGTCAC<br>GTAGTGCCTGGTCAGTTCATC	59.2	287
β-Actin	GTACCACAGGCATTGTGATG<br>GAGAAGCTGTGCTATGTTGC	53.2	219

[1] M. Marti, L. Mulero, C. Pardo et al., "Characterization of pluripotent stem cells", *Nature protocols,* vol. 8, no.2, pp. 223-253, 2013.
